# Conformational Control of Dual Emission by Pyrrolidinyl PNA–DNA Hybrids

**DOI:** 10.1002/open.201200016

**Published:** 2012-07-11

**Authors:** Sabrina Sezi, Reji Varghese, Tirayut Vilaivan, Hans-Achim Wagenknecht

**Affiliations:** a Institute for Organic Chemistry, Karlsruhe Institute of TechnologyFritz-Haber-Weg 6, 76131 Karlsruhe (Germany) E-mail: Wagenknecht@kit.edu; b Department of Chemistry, Faculty of Science, Chulalongkorn UniversityPhyathai Road, Pathumwan, Bangkok 10330 (Thailand) E-mail: vtirayut@chula.ac.th

**Keywords:** biosensors, fluorescence, nanomaterials, nucleic acids, peptides

The current interest in using nucleic acids as supramolecular scaffolds for the precise arrangement of chromophores is extremely high.^1^ This is triggered by the potential to control chromophore interactions through the geometry of the double helical structure and is also driven by the search for new multifluorescent probes for bioanalytics and for new multiluminescent organic materials.^2^–^6^ However, the construction of nucleic acid-based multichromophores requires the understanding of photophysical interactions in chromophore pairs as the simplest unit along the helix. Wilhemsson et al. used rigid nucleic acid base analogues to study the influence of the relative orientation on energy transfer.^6^ Recently, we published on white light-emitting DNA by combining ethynyl pyrene and ethynyl nile red as blue-green and red emitters.^4^ Herein, we extend this concept to pyrrolidinyl PNA–DNA hybrids to study the influence of a conformationally restricted nucleic acid geometry.

Peptide nucleic acid (PNA) is a class of DNA analogues that consists of nucleobases attached to a peptide-like backbone. The original PNA with a flexible *N*-(2-aminoethyl)glycine backbone introduced by Nielsen et al. was shown to bind strongly to DNA and preferentially to RNA with high affinity and sequence specificity.^7^, ^8^ We,^9^–^11^ as well as others,^12^–^14^ have shown that incorporating conformational rigidity into the PNA backbone could further improve the binding affinity, especially to DNA, as a result of decreased entropy loss upon hybridization. PNA systems derived from nucleobase-modified proline in (2′*R*,4′*R*) configurations in combination with cyclic β-amino acids including (*R*)-aminopyrrolidine-2-carboxylic acid (dapcPNA),^9^ (2*S*)-aminocyclopentane-(1*S*)-carboxylic acid (acpcPNA)^10^ and (2*S*)-aminocyclobutane-(1*S*)-carboxylic acid (acbcPNA)^11^ formed very stable hybrids with complementary DNA. Much less stable hybrids were formed with RNA, and no self-pairing hybrids could be obtained in acpcPNA.^10^ These properties have not been observed in other PNA systems. Recently, the artificial nucleobase 5-(1-pyrenyl)uracil was incorporated into acpcPNA to probe the sequence selectivity by fluorescence of hybrids with DNA.^15^

We applied, herein, dual-emitting oligonucleotides bearing ethynyl pyrene adjacent to ethynyl nile red to probe the differences in interactions in DNA duplexes compared to conformationally constrained acpcPNA–DNA hybrids. Using our published synthetic protocols,^4^, ^10^ we prepared doubly labeled oligonucleotides **DNA1** to **DNA4** and the corresponding acpcPNA counterstrands, **PNA1** to **PNA4**. These strands can form fully complementary DNA–PNA hybrids in the binding region (e.g., **DNA1**–**PNA1**) in which the two fluorophores are embedded in four different sequential contexts (X- - - -Y, Scheme 1). **DNA5** to **DNA8** represent oligonucleotide counterstrands to **DNA1** to **DNA4**, respectively, in order to compare the optical properties of the PNA–DNA hybrids with the corresponding doubly modified DNA duplexes (e.g., **DNA1**–**DNA5**). Additionally, PNA–DNA hybrids or DNA duplexes with two mismatches can be formed (e.g., **DNA1**–**PNA2** or **DNA1**–**DNA6**). **DNA9** to **DNA12** represent reference oligonucleotides to measure the thermal stability of the unmodified acpcPNA–DNA hybrids (e.g,. **DNA9**–**PNA1**).

**Scheme 1:**
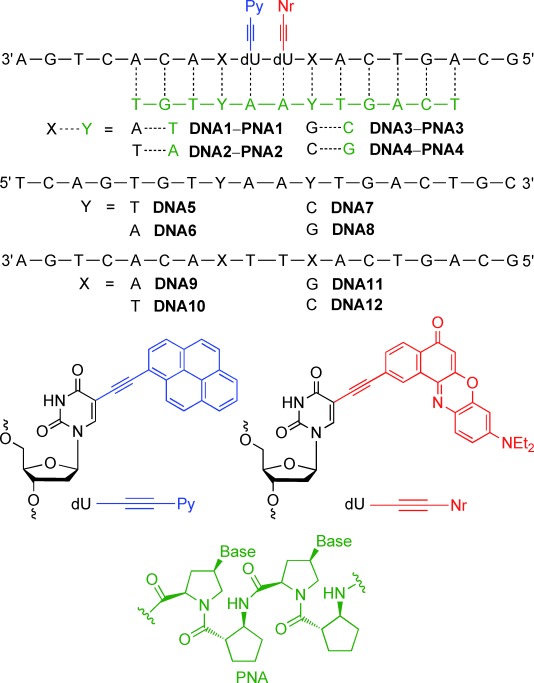
Sequences of modified oligonucleotides **DNA1** to **DNA4**, acpcPNA counterstrands **PNA1** to **PNA4**, oligonucleotide counterstrands **DNA5** to **DNA8** and reference oligonucleotides **DNA9** to **DNA12**.

The optical properties of the acpcPNA–DNA hybrids in comparison with the corresponding DNA duplexes were characterized by UV/Vis absorption spectra, including melting temperatures (*T*_m_), and steady-state fluorescence spectroscopy (Table 1). The unmodified hybrids **DNA9**–**PNA1** to **DNA12**–**PNA4** exhibit *T*_m_ values ranging from 71.8 °C to >90 °C. Considering the fact that the PNA counterstrands **PNA1** to **PNA4** represent only a partial, 12 bases long complement, these *T*_m_ values are extremely high and show the extraordinary stabilizing effect of acpcPNA.^10^ On the other hand, it is highly interesting that the thermal stabilities of the acpcPNA–DNA hybrids contradict the known sequential effects in normal DNA. The only difference is that **DNA9**–**PNA1** and **DNA10**–**PNA2** contain two A-T base pairs where **DNA11**–**PNA3** and **DNA12**–**PNA4** bear two G-C base pairs. In normal DNA, an exchange of A-T by G-C base pairs increases the *T*_m_ values. With acpcPNA, the opposite effect is obtained, and the thermal stability is enhanced with A-T base pairs. The *T*_m_ values of **PNA1** and **PNA3** with their exactly complementary, unmodified DNA targets were 85.4 and 71.0 °C, respectively. These values are comparable to those obtained from the long target sequences used in this work, indicating that the binding should occur at the expected region. All four doubly labeled hybrids, **DNA1**–**PNA1** to **DNA4**–**PNA4**, show reduced *T*_m_ values of 58.2–82.3 °C, due to the presence of the two fluorophores. On the other hand, the *T*_m_ values of these acpcPNA–DNA hybrids are generally higher compared with the corresponding doubly labeled DNA duplexes (i.e., **DNA1**–**DNA5** to **DNA4**–**DNA8,** except **DNA3**–**DNA7** in comparison with **DNA3**–**PNA3**). Mismatch-containing DNA duplexes (e.g., **DNA1**–**DNA6**) show strongly reduced thermal stabilities (−9.3 °C to −27.9 °C), while mismatch-containing acpcPNA–DNA hybrids are not formed at all (e.g. **DNA1**–**PNA2**), as expected based on our previous results and as supported by the low absorption differences in the melting temperature experiments.^10^

**Table 1:** Melting temperatures (*T*_m_) and optical properties of acpcPNA–DNA hybrids in comparison with DNA duplexes

Sequence[a]		*T*_m_[b] [°C]	Δ*A*_402_/Δ*A*_379_[c]	*λ* [nm][d]	*I*_665_/*I*_440_[e]
**DNA1**	–	–	0.97	621	8.9
**DNA1–DNA5**	m	58.5	0.99	622	2.7
**DNA1–DNA6**	mm	48.7	1.02	624	9.0
**DNA1–PNA1**	m	82.3	0.92	618	3.6
**DNA1–PNA2**	mm	–[f]	0.96	622	9.1
**DNA9–PNA1**	m	86.0	–	–	–
					
**DNA2**	–	–	0.94	618	>10
**DNA2–DNA6**	m	57.2	0.97	620	4.3
**DNA2–DNA5**	mm	47.9	0.99	618	11.5
**DNA2–PNA2**	m	76.5	0.90	631	1.2
**DNA2–PNA1**	mm	–[f]	0.96	619	6.3
**DNA10–PNA2**	m	>90	–	–	–
					
**DNA3**		–	1.03	621	8.5
**DNA3–DNA7**	m	63.5	1.01	621	5.2
**DNA3–DNA8**	mm	51.2	1.06	622	>10
**DNA3-PNA3**	m	58.2	0.95	623	2.2
**DNA3–PNA4**	mm	–[f]	1.03	621	7.4
**DNA11–PNA3**	m	71.8	–	–	–
					
**DNA4**	–	–	0.91	622	>10
**DNA4–DNA8**	m	64.3	0.98	623	1.7
**DNA4–DNA7**	mm	36.4	0.91	621	>10
**DNA4–PNA4**	m	77.4	0.90	637	1.0
**DNA4–PNA3**	mm	–[f]	0.91	622	8.9
**DNA12–PNA4**	m	>90	–	–	–

[a] m=match, mm=mismatch.

[b] *Conditions:* duplex (2.5 μm), buffer (10 mM Na-P_i_, 250 mM NaCl, pH 7), 20–90 °C, 0.7 °C min^−1^.

[c] Absorption ratios at 402 nm versus 379 nm.

[d] Absorption maximum of the nile red chromophore.

[e] Fluorescence intensity ratios at 665 nm versus 440 nm.

[f] ΔΔ*A*<0.05.

The UV/Vis absorption of both the doubly modified acpcPNA–DNA hybrids and the DNA duplexes show the presence of both chromophores at ∼370–400 nm (ethynyl pyrene) and ∼620 nm (ethynyl nile red). If excited at 380 nm, which is highly selective for ethynyl pyrene, the various single strands, hybrids and duplexes show dual emission at ∼440 nm (ethynyl pyrene) and ∼665 nm (ethynyl nile red) as the result of an energy transfer between both chromophores. In the most interesting case from the supramolecular point of view, the contributions of the blue-green and the red emission are equal, and thereby an emission of white light is generated.^4^ A careful examination reveals two major differences in the UV/Vis absorption of the various samples (Figure 1 and the Supporting Information): Both the ratio of the two absorption bands of ethynyl pyrene at ∼402 nm and ∼379 nm as well as the maximum of nile red absorption varies significantly (Table 1). According to our previous results, the UV/Vis band at approximately 402 nm represents the absorption of an excitonically coupled complex of the ethynyl pyrene chromophore with one of the adjacent DNA bases.^16^ In the randomly folded single stranded DNA, these interactions are generally less pronounced. However, a higher extent of prestacking between the ethynyl pyrene and purines (A or G) exist in **DNA1** and **DNA3**, respectively, as the corresponding absorption ratios (Δ*A*_402_/Δ*A*_379_) are higher compared with those of **DNA2** and **DNA4**. In principle, ground state interactions interfere with energy transfer, because the latter process requires the excitation of an uncoupled chromophore as energy donor (ethynyl pyrene) and subsequent transfer of excitation energy to an uncoupled and unexcited chromophore as acceptor (ethynyl nile red). Hence, it is not surprising that those single stranded oligonucleotides (**DNA2** and **DNA4**), in which the ethynyl pyrene is less coupled in the ground state (according to the ratios Δ*A*_402_/Δ*A*_379_), exhibit better energy-transfer efficiencies (indicated by higher nile red emission, and displayed by higher fluorescence intensity ratios *I*_665_/*I*_440_>10).

**Figure 1:**
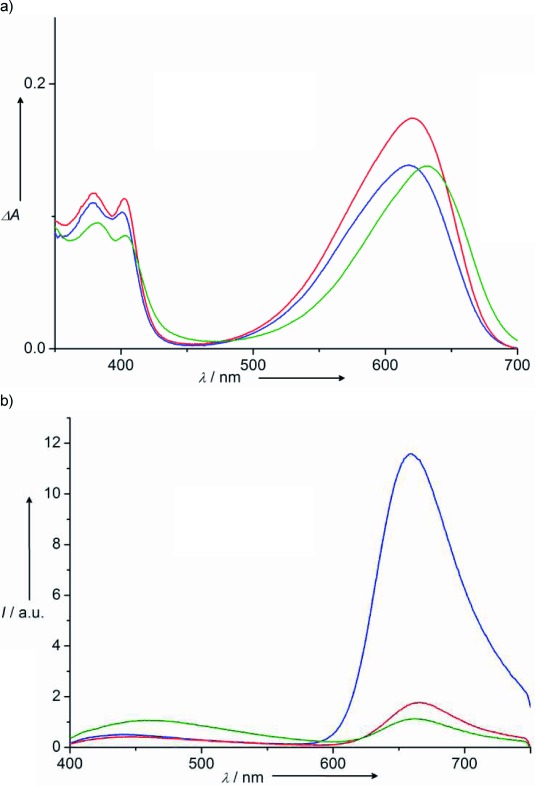
a) UV/Vis absorption and b) fluorescence representatively shown for single stranded **DNA2** (—), double stranded **DNA2**–**DNA6** (—) and **DNA2**–**PNA2** (—; 2.5 μM each) in buffer (10 mM Na-P_i_, 250 mM NaCl, pH 7) at 20 °C and *λ*_exc_ 380 nm.

If the single strands **DNA1** to **DNA4** are annealed with the corresponding fully complementary counterstrands **DNA5** to **DNA8**, the absorption side band at ∼402 nm gets intensified, and the ratios Δ*A*_402_/Δ*A*_379_ increase accordingly. This is not a new result and has been reported previously.^14^ However, intensified ground state interactions of the ethynyl pyrene chromophore with adjacent DNA bases in double stranded DNA inhibits energy transfer to ethynyl nile red. As a result, the fluorescence intensity at ∼665 nm gets decreased significantly *without* a corresponding increase of the ethynyl pyrene emission at ∼440 nm. The ratio of the fluorescence intensities *I*_665_/*I*_440_ are reduced to values between 1.7 (**DNA4**–**DNA8**) and 5.2 (**DNA3**–**DNA7**). It is important to note here that the excitonic interactions do not require the fully matched DNA architecture around them, as they are formed also in the mismatched DNA duplexes (**DNA1**–**DNA6** to **DNA4**–**DNA7**). However, in the latter duplexes they do not interfere with energy transfer. Obviously, transition dipole moments of the nonstacked subensemble can—due to higher conformational flexibility—adapt an orientation that is more suitable for efficient energy transfer. Thus, fluorescence intensity ratios *I*_665_/*I*_440_ in the mismatched duplexes are similar to those of the corresponding single strands **DNA1** to **DNA4**.

If the single strands **DNA1** to **DNA4** are annealed with corresponding fully complementary acpcPNA strands **PNA1** to **PNA4**, the optical properties change quite significantly compared with the DNA duplexes. Except for **DNA1**–**PNA1**, the *I*_665_/*I*_440_ ratios are generally lower than that of the corresponding DNA duplexes, ranging between 1.0 (**DNA4**–**PNA4**) to 3.6 (**DNA1**–**PNA1**). The fluorescence intensity ratio bears no relationship to the duplex stability as can be seen by the lower *I*_665_/*I*_440_ ratio, despite the lower thermal stability of **DNA3**–**PNA3** compared with **DNA3**–**DNA7**. In addition, the absorption side bands of ethynyl pyrene at ∼402 nm get reduced and the absorption ratios Δ*A*_402_/Δ*A*_379_ get decreased accordingly. This observation indicates that the stacking interactions between the ethynyl pyrene chromophore and adjacent DNA bases are interrupted by the conformational constrain, which is introduced in acpcPNA–DNA hybrids. According to our discussion for the matched DNA duplexes above, this should yield stronger nile red fluorescence intensity at ∼665 nm compared with DNA double strands. However, the opposite effect is observed: the red emission is also reduced in the acpcPNA–DNA hybrids. This result can be explained by two different effects that are probably combined to various extents in the different acpcPNA–DNA hybrids. Firstly, except for **DNA1**–**PNA1**, the ethynyl pyrene fluorescence in acpcPNA–DNA hybrids increases slightly relative to DNA duplexes. The observed difference of the energy-transfer efficiencies is likely due to the change in the relative orientation of the chromophore dipole moments^3^ upon duplex formation with PNA (helically twisted conformation), which partially prohibits an efficient energy transfer. With the published doubly modified DNA–DNA duplexes^4^ we observed that even if the difference in the energy transfer is small (according to the rather small increase of ethynyl pyrene fluorescence intensity), the significant drop of the red emission intensity in the duplex is due to the large molar extinction coefficient and the high fluorescence quantum yield of ethynyl nile red. Secondly, a careful examination of the ethynyl nile red absorption reveals changes of its maximum wavelength (most pronounced in **DNA2**–**PNA2**). This again supports ground state interactions between nile red and ethynyl pyrene, and thereby again interferes with ethynyl nile red emission induced by energy transfer.

The inspection of photophysical interactions in chromophore pairs is crucial for using the geometries of nucleic acid hybrids with artificial backbones for the development of multichromophore systems. From our results it becomes clear that the strong binding to DNA (high *T*_m_ values) and the pronounced sequence selectivity including mismatch discrimination of acpcPNA provides added value to the basic concept of self-assembled nucleic acid-based nanostructures and multichromophore arrangements. Among the samples presented herein, especially hybrids **DNA2**–**PNA2** and **DNA4**–**PNA4** show nearly equal contributions of the blue-green and red emission and, thus, represent white-light emitters with better thermal stability than the previously published white light-emitting DNA (WED).^4^ These results demonstrate that the control of photophysical interactions between chromophores can be much better realized with the sterically constrained pyrrolidinyl PNA. Especially acpcPNA has a significant potential and should play an increasing role for the development of both fluorescent probes and nucleic acid-based nanomaterials.

## Supplementary material

Detailed facts of importance to specialist readers are published as ”Supporting Information”. Such documents are peer-reviewed, but not copy-edited or typeset. They are made available as submitted by the authors.
