# Supplementary material for: Conformational Control of Dual Emission by Pyrrolidinyl PNA–DNA Hybrids
Source: ChemistryOpen. 2012 Jul 11;1(4):173–6. doi: 10.1002/open.201200016 (PMC3922446; doi:10.1002/open.201200016)
Supplement: Supplementary file 1 [file open0001-0173-SD1.pdf]

ChemistryOPEN

Supporting Information

© Copyright Wiley-VCH Verlag GmbH & Co. KGaA, 69451 Weinheim, 2012

Conformational Control of Dual Emission by Pyrrolidinyl PNA–DNA Hybrids

Sabrina Sezi,[a] Reji Varghese,[a] Tirayut Vilaivan,*[b] and Hans-Achim Wagenknecht*[a]

open_201200016_sm_miscellaneous_information.pdf

Materials and Methods

Chemicals and dry solvents were purchased from commercial suppliers and were used without further purification unless otherwise mentioned. Spectroscopic measurements were recorded in Na-P*i* buffer solution (10 mM, pH = 7) using quartz glass cuvettes (*l* = 10 mm). ESI mass spectra were measured in the central analytical facility of the Faculty of Chemistry and Pharmacy at the University of Regensburg on a ThermoQuest Finnigan TSQ 7000 in negative ionisation mode. MALDI-TOF mass spectra were measured at the Karlsruhe Institute of Technology on a BIFLEX-IV spectrometer (Bruker Daltonics) in linear negative mode. A 2:1-mixture (v/v) of 2',4',6'-Trihydroxyacetophenone (THAP; 0.3 M in EtOH) and ammonium citrate (dibasic; 0.1 M in H_2O) was used as matrix. The identity of each PNA was verified by MALDI-TOF mass spectrometry (Bruker Daltonik GmbH, Germany) in linear positive mode using α-cyano-4-hydroxycinnamic acid (CCA) matrix.

Absorption spectra and melting temperatures (2.5 μM DNA, 20-90 °C, 0.7 °C/min, step width 0.5 °C) were recorded on a Varian Cary 100 spectrometer equipped with a 6×6 cell changer unit. Fluorescence was measured on a Jobin-Yvon Fluoromax 3 fluorimeter with a step width of 1 nm and an integration time of 0.2 s. All spectra were recorded with an excitation and emission bandpass of 5 nm and are corrected for Raman emission from the buffer solution.

Synthesis of DNA1 to DNA4

The oligonucleotides were prepared on an Expedite 8909 DNA synthesizer (Applied Biosystems) via standard phosphoramidite protocols using CPGs (1 μmol) with a longer coupling time of 30 minutes and a higher concentration of the nile red phosphoramidite (0.1 M). The chemicals for the DNA synthesis were purchased from ABI and Glen Research. After preparation, the trityl-off oligonucleotide was cleaved off the resin and was deprotected by treatment with conc. NH_4OH at 45°C for 16 h. The oligonucleotides were dried and purified by reverse phase HPLC using the following conditions: A = NH_4OAc buffer (50 mM), pH = 6.5; B = MeCN; gradient = 0-30% B over 50 min. The oligonucleotides were lyophilised and quantified by their absorbance at 260 nm. Duplexes were

prepared by heating the chromophore-modified (**DNA1 to DNA4**) or unmodified (**DNA9 to DNA12**) oligonucleotides in the presence of 1.2 equiv. unmodified complementary strand (**PNA1 to PNA4** or **DNA5 to DNA8**) to 90 °C (hold for 10 min), followed by slow cooling to r.t.

Synthesis of PNA1 to PNA4

The PNAs were synthesized manually by Fmoc-solid phase peptide synthesis on TentaGel S RAM resin (0.24 mmol/g substitution, 1.5 μmol) preloaded with Fmoc-L-Lys(Mmt). After the last synthesis cycle, the N-termini were capped by benzoylation (**PNA1, PNA2 and PNA4**) or acetylation (**PNA3**). The nucleobase protecting groups (Bz and Ibu) were removed by 1:1 aqueous NH_3:dioxane at 60 ºC overnight. Cleavage of the PNA oligomers from the resin was achieved by treatment with trifluoroacetic acid (TFA). After precipitation with diethyl ether, the PNAs were purified by reverse phase HPLC using the following conditions: A = 0.1% TFA in water; B = 0.1% TFA in MeOH; gradient = 10-90% B over 30 min. The collected fractions were lyophilised and quantified by their absorbance at 260 nm.

Table S1. *m/z* Values of ss-**DNA1** to **DNA4** as determined by ESI-MS / MALDI-TOF-MS.

**DNA**	Calculated mass (*m/z*)	Observed mass (*m/z*)
**DNA1**	6025.9	6030.0
**DNA2**	6007.9	6012.4
**DNA3**	6059.9	6060.9
**DNA4**	5977.9	5981.8

Table S2. *m/z* Values of ss-**PNA1** to **PNA4** as determined by MALDI-TOF-MS.

**PNA**	Calculated mass (*m/z*)	Observed mass (*m/z*)
**PNA1**	4300.6	4300.7
**PNA2**	4318.7	4319.2
**PNA3**	4208.5	4207.0
**PNA4**	4350.7	4349.9

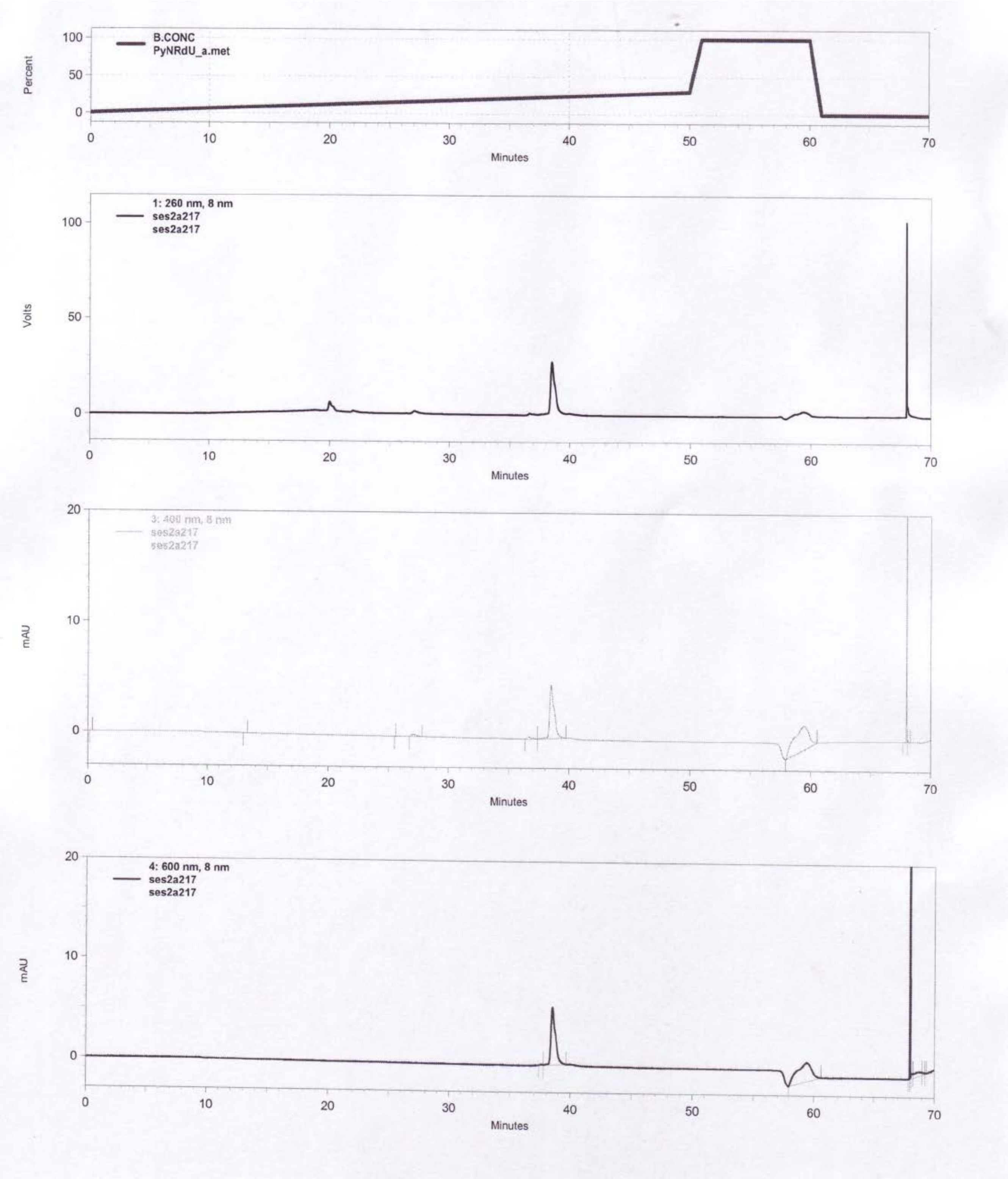

Figure S1. Image of analytical HPLC traces of **DNA1**.

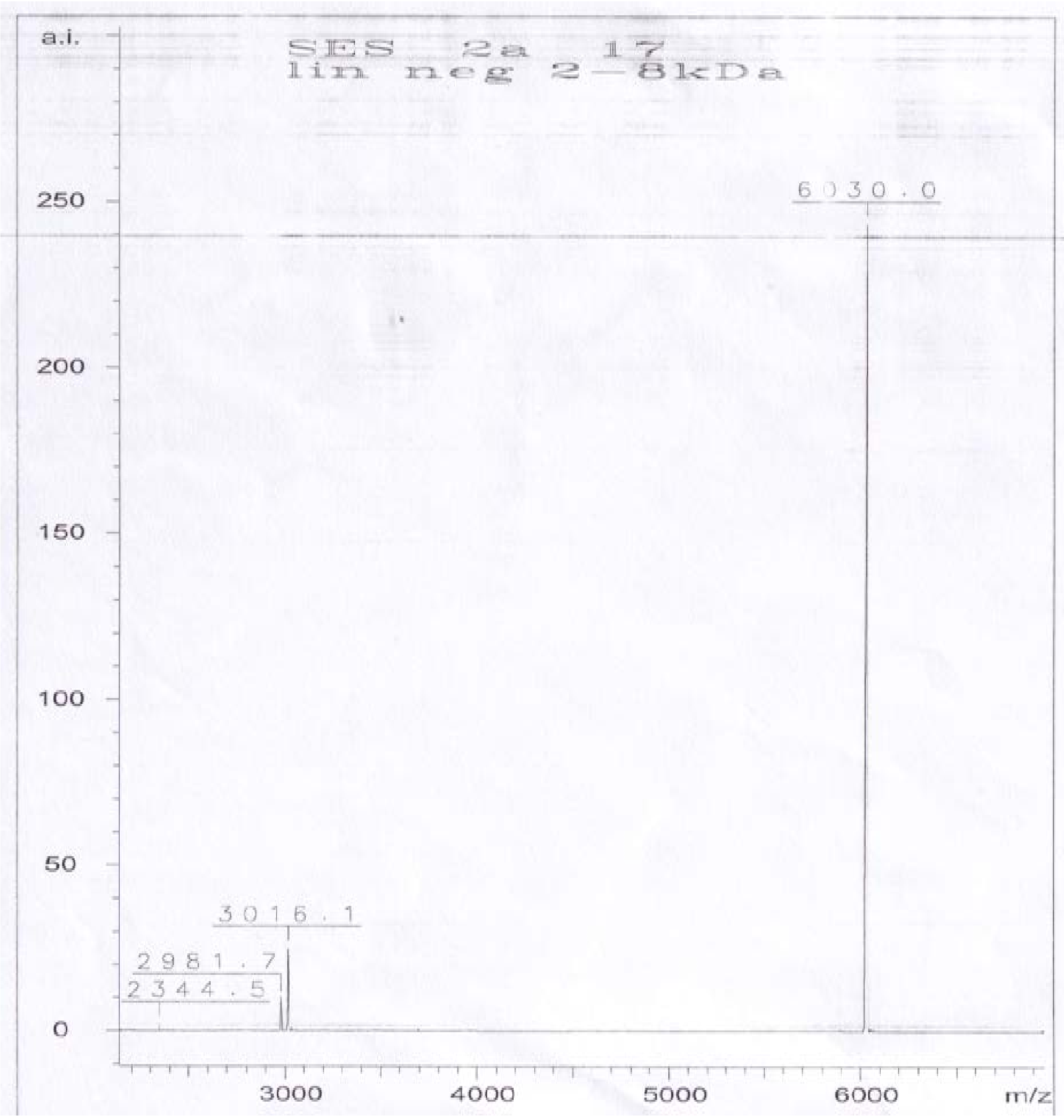

Figure S2. Image of MALDI-TOF mass spectrum of **DNA1**.

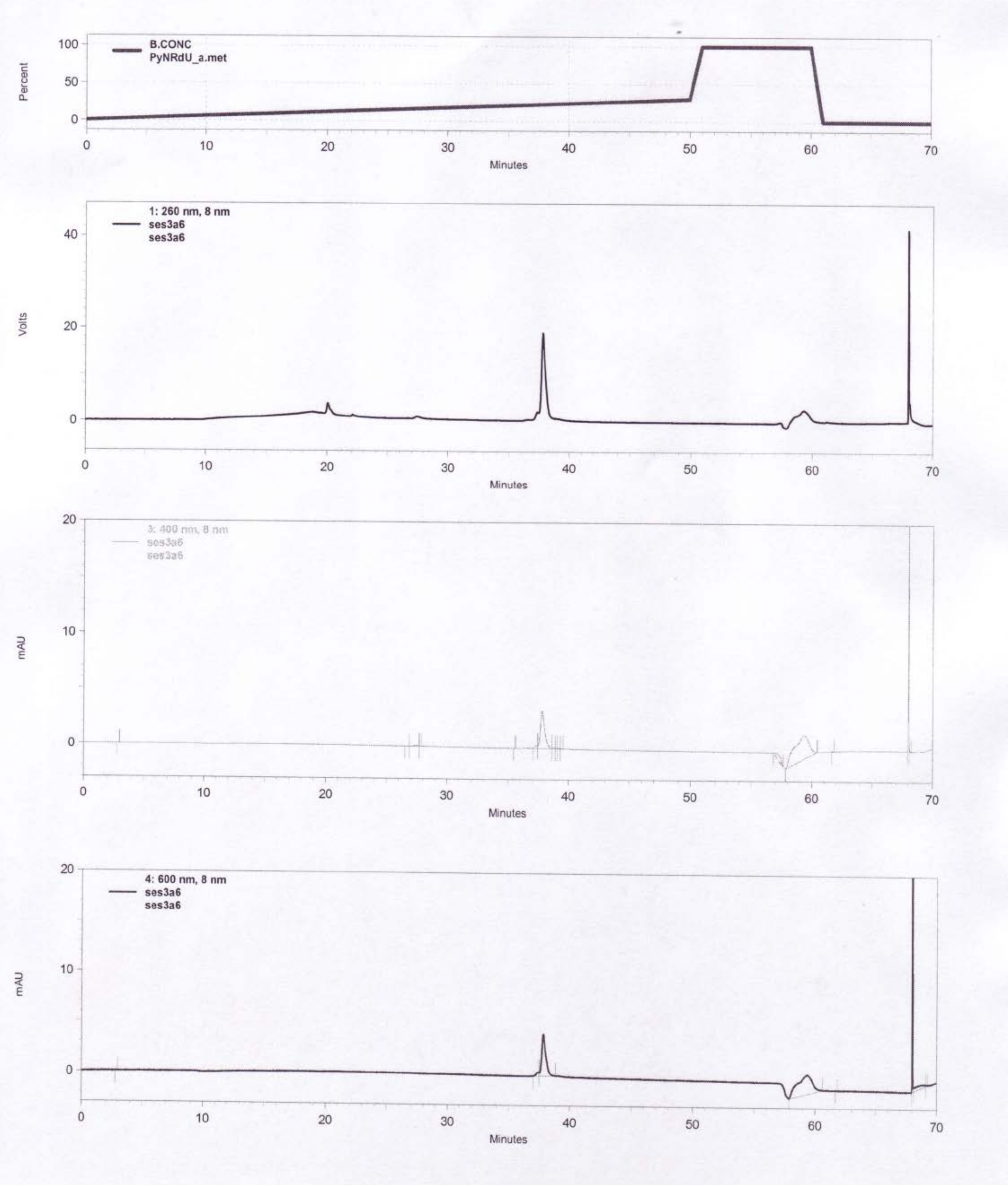

Figure S3. Image of analytical HPLC traces of **DNA2**.

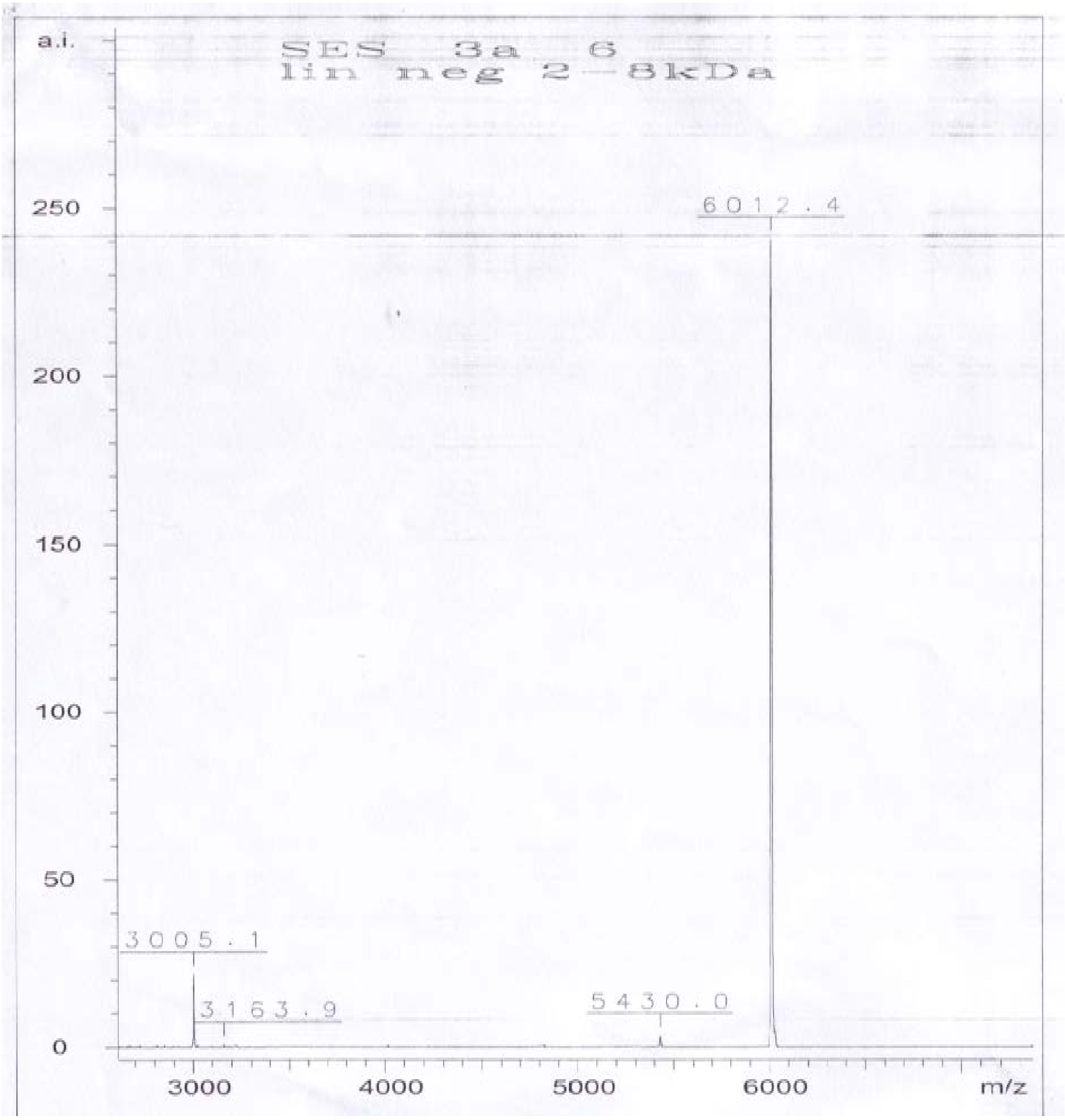

Figure S4. Image of MALDI-TOF mass spectrum of **DNA2**.

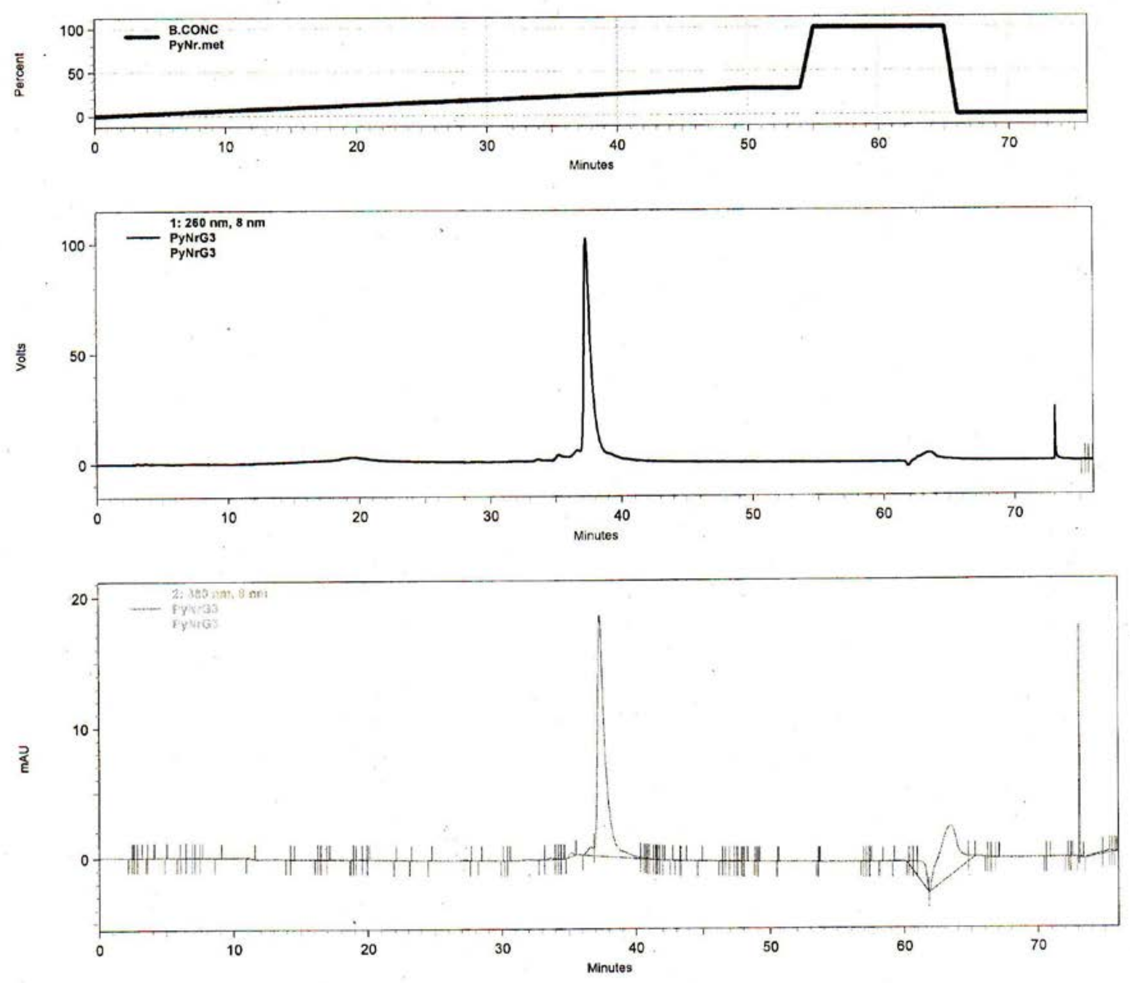

Figure S5. Image of analytical HPLC traces of **DNA3.**

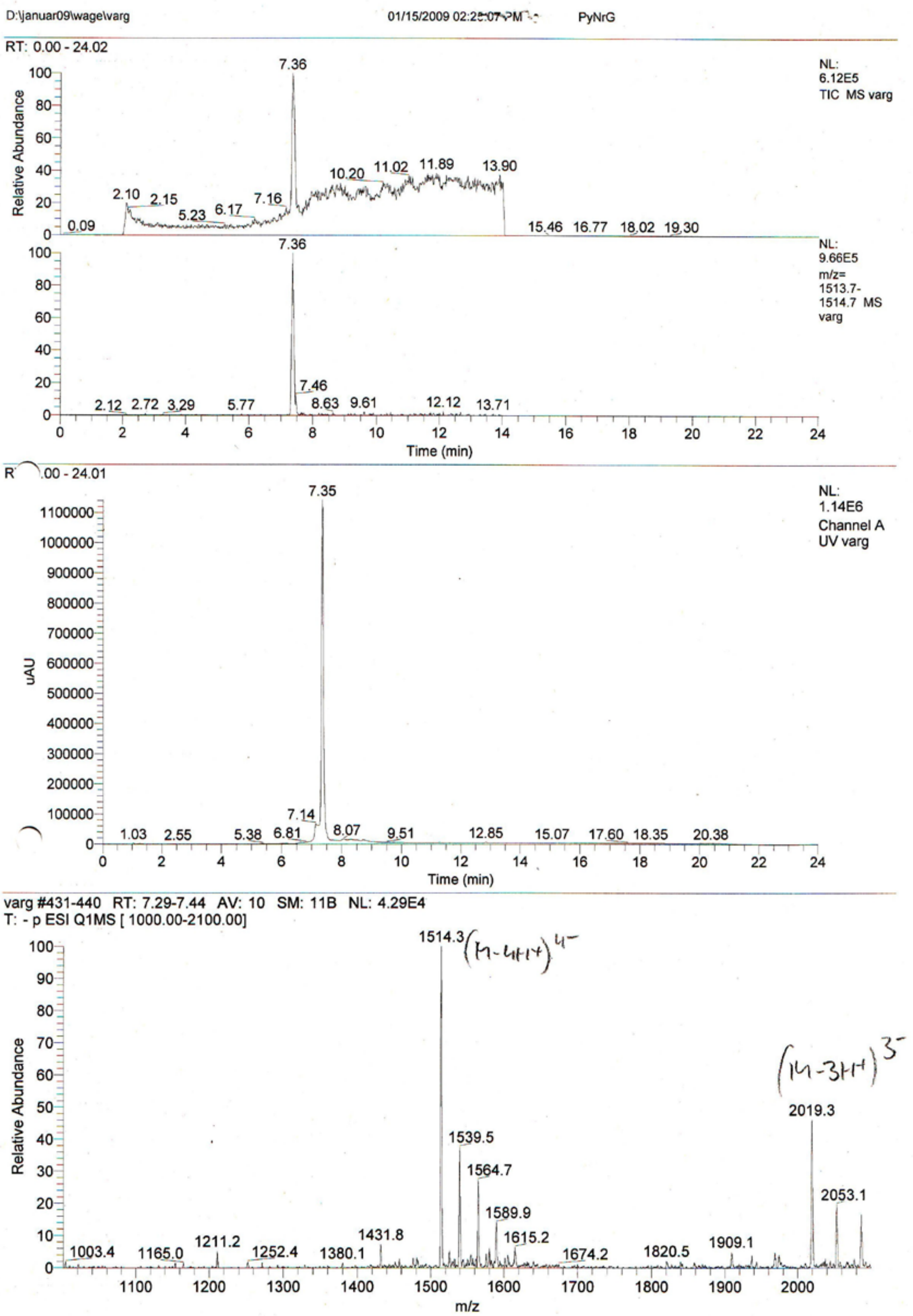

Figure S6. Image of LC-ESI-MS of **DNA3**.

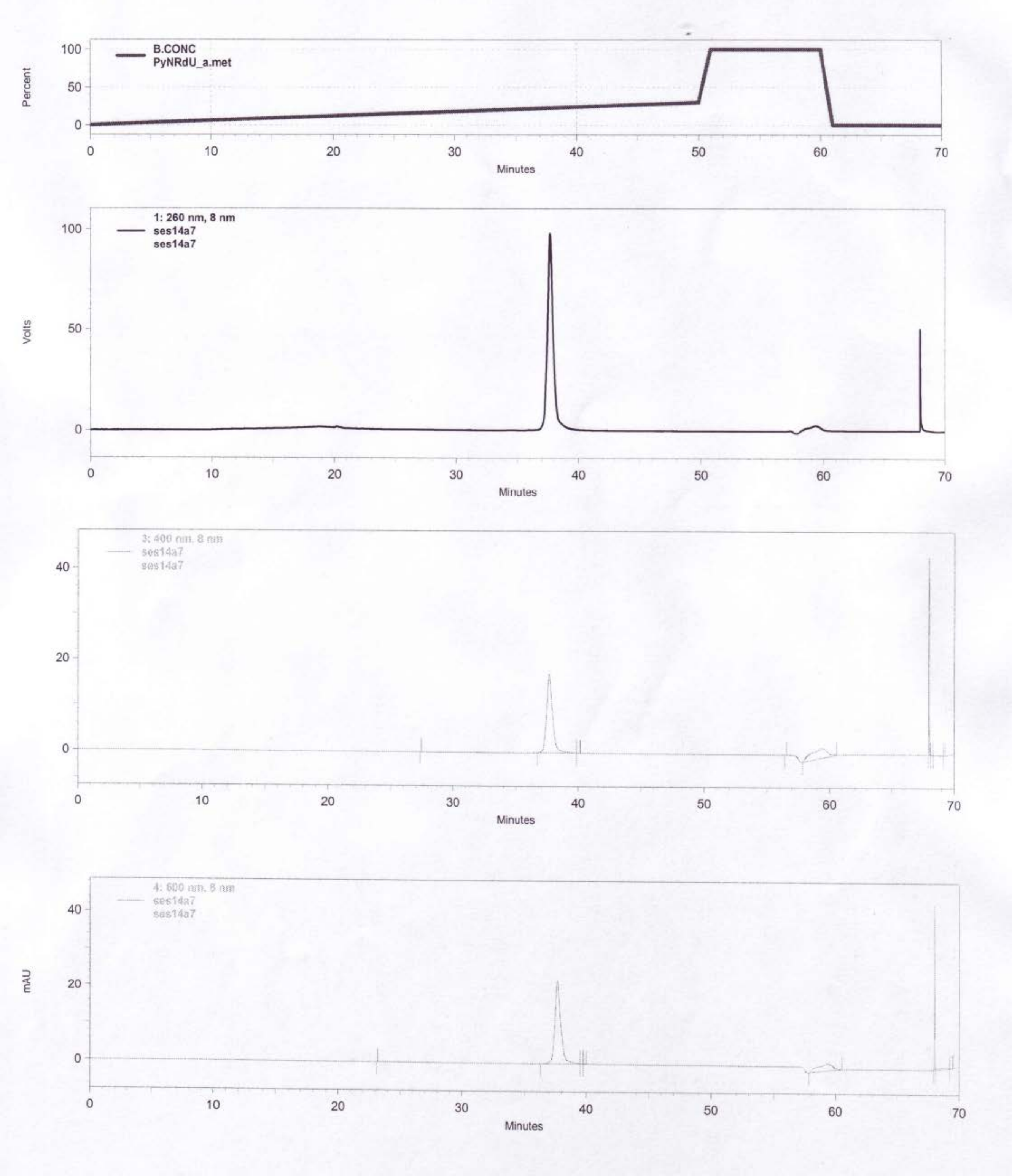

Figure S7. Image of analytical HPLC traces of **DNA4.**

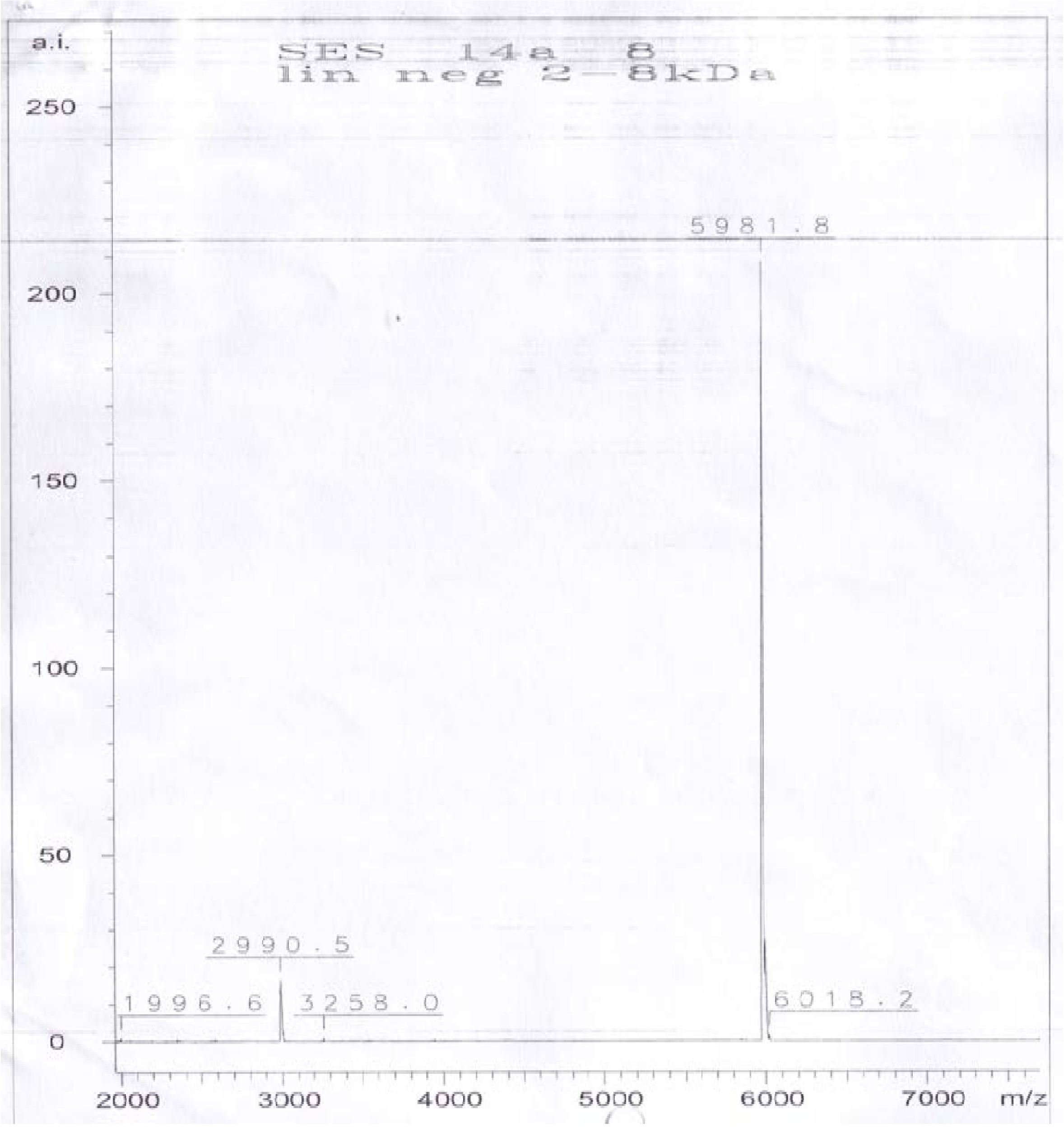

Figure S8. Image of MALDI-TOF mass spectrum of **DNA4**.

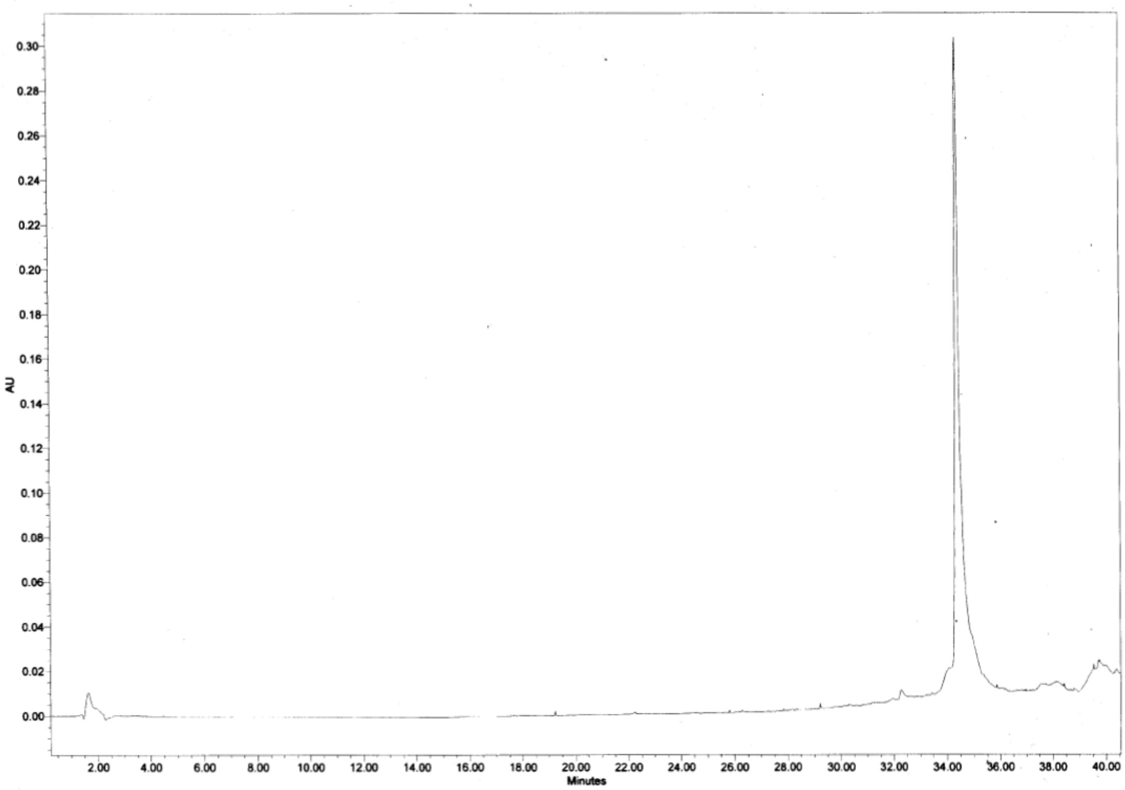

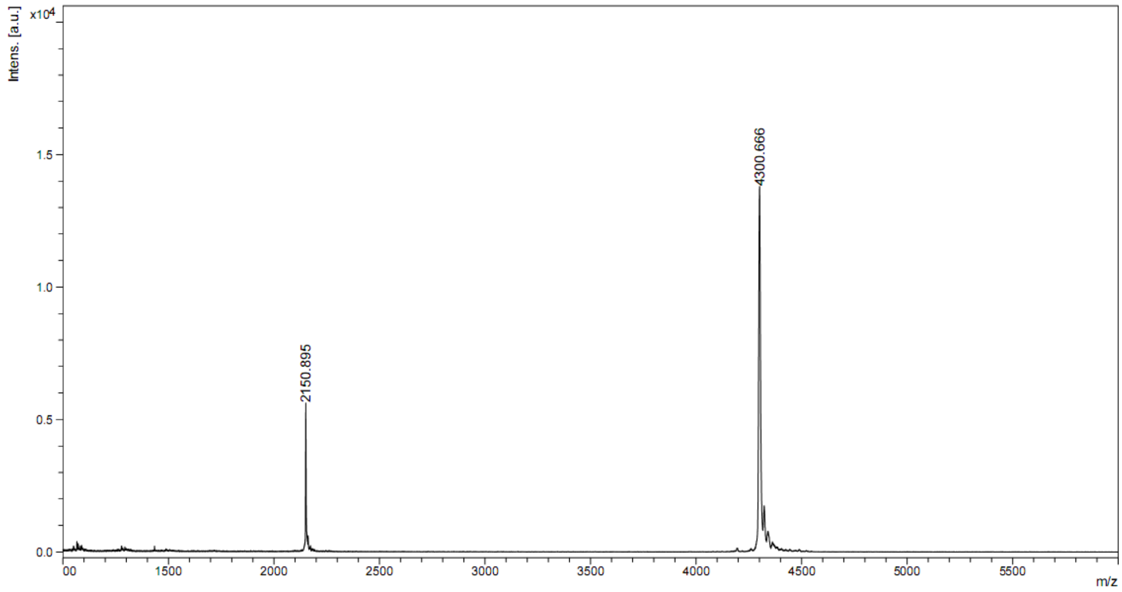

Figure S9. Image of analytical HPLC traces (top) and MALDI-TOF mass spectrum (bottom) of **PNA1**.

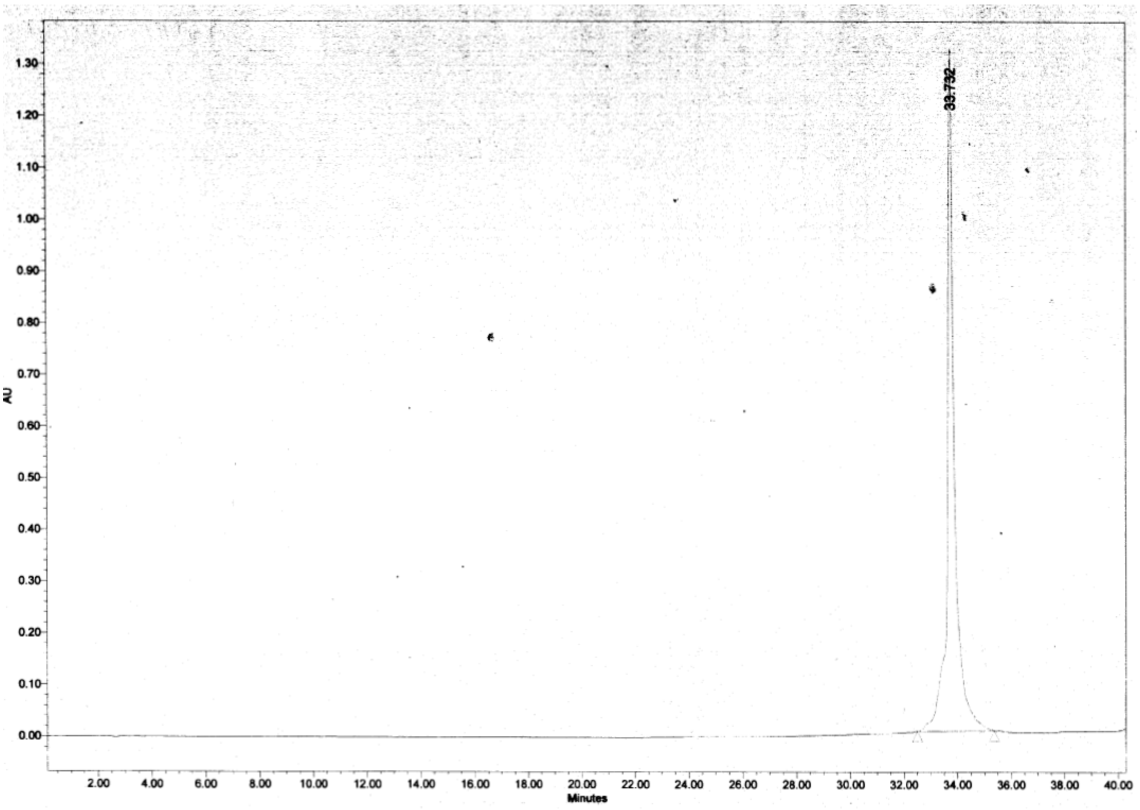

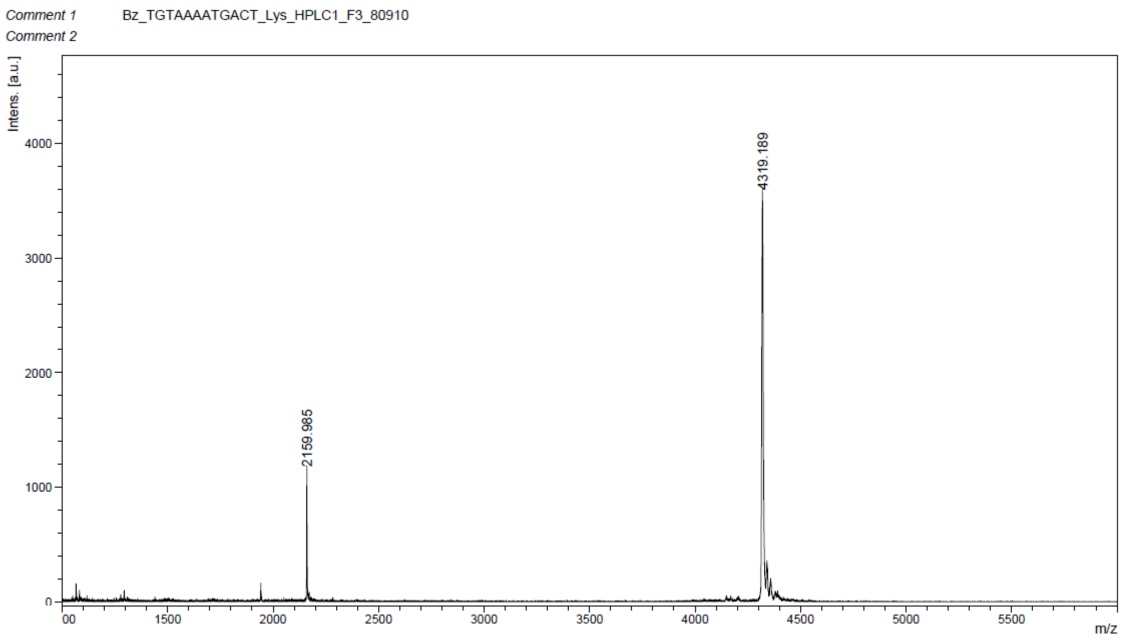

Figure S10. Image of analytical HPLC traces (top) and MALDI-TOF mass spectrum (bottom) of **PNA2**

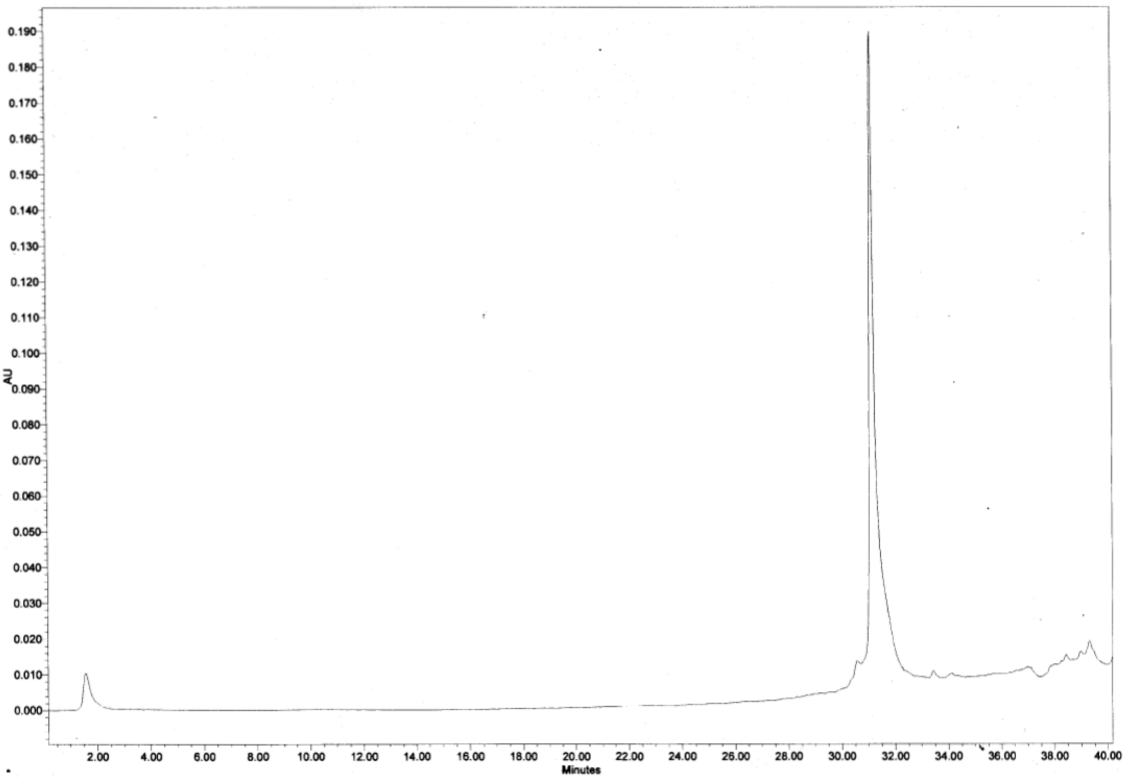

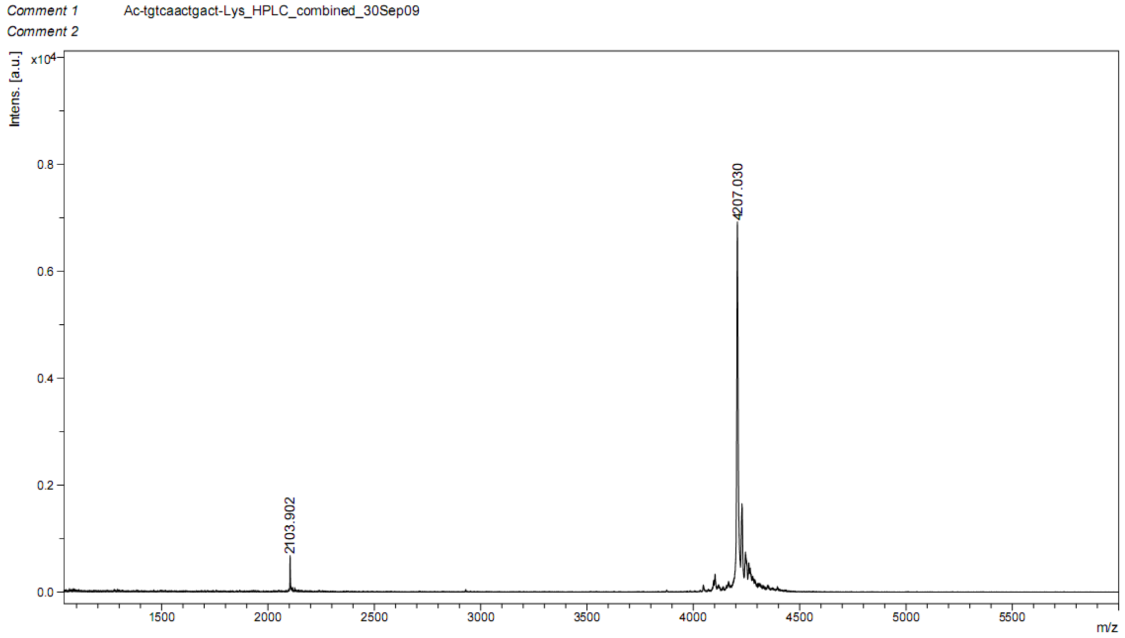

Figure S11. Image of analytical HPLC traces (top) and MALDI-TOF mass spectrum (bottom) of **PNA3**.

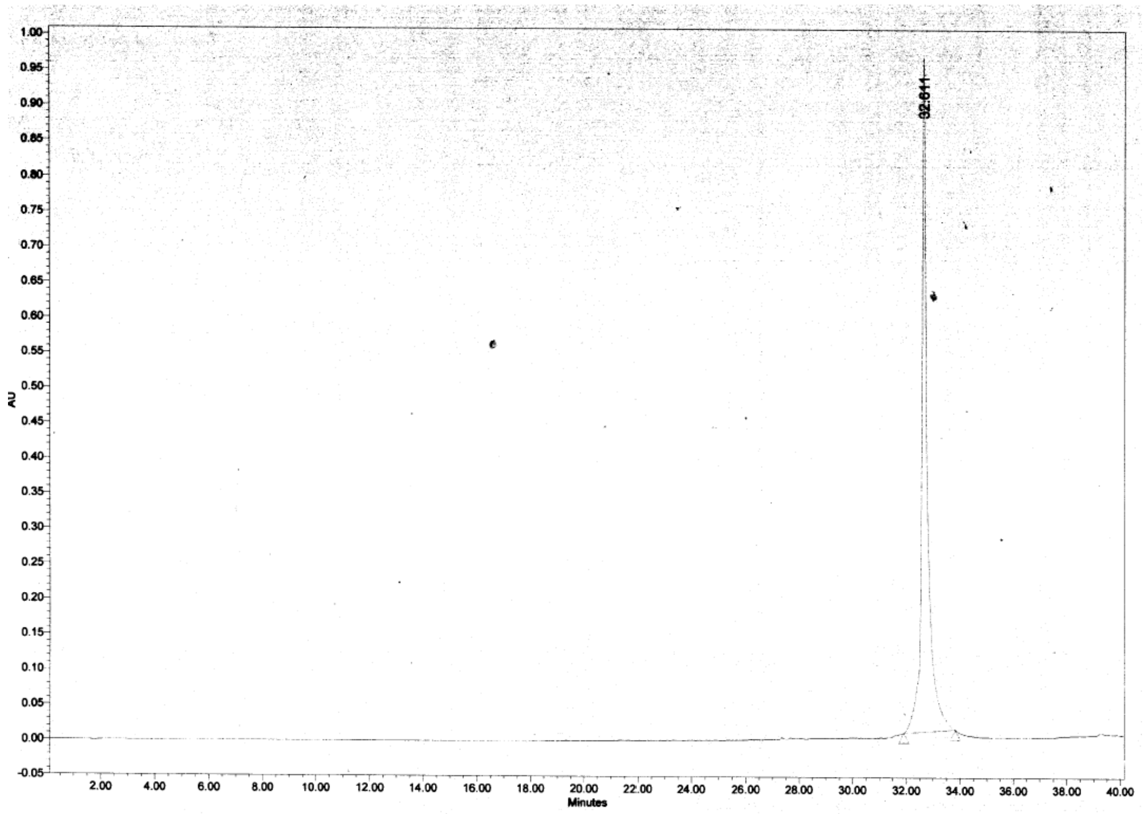

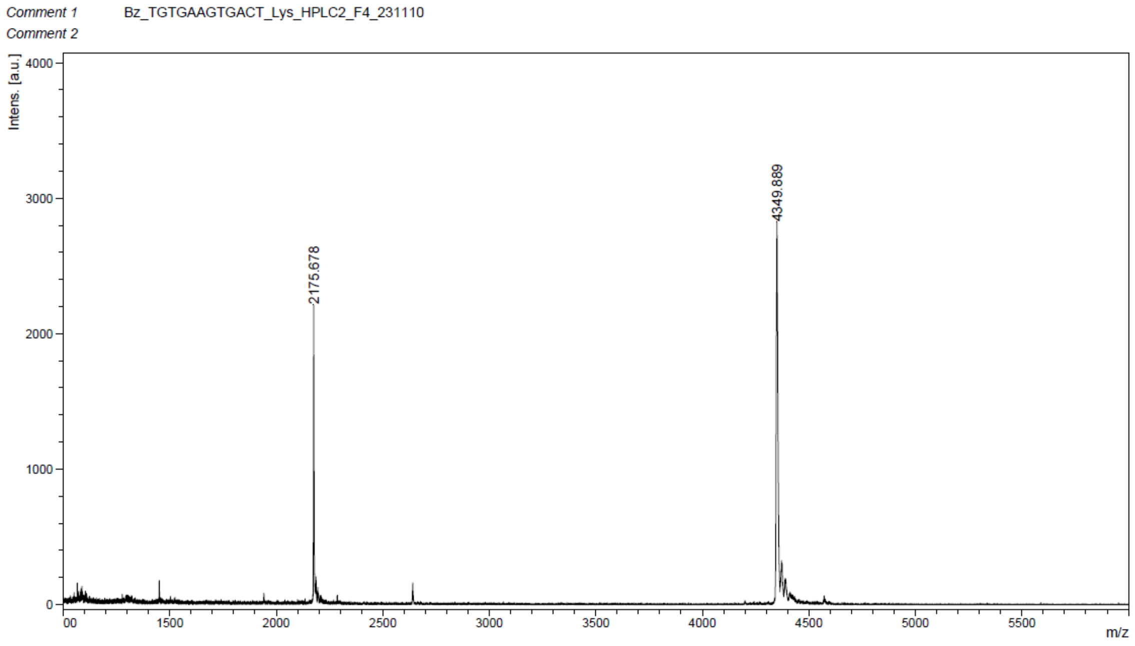

Figure S12. Image of analytical HPLC traces (top) and MALDI-TOF mass spectrum (bottom) of **PNA4**

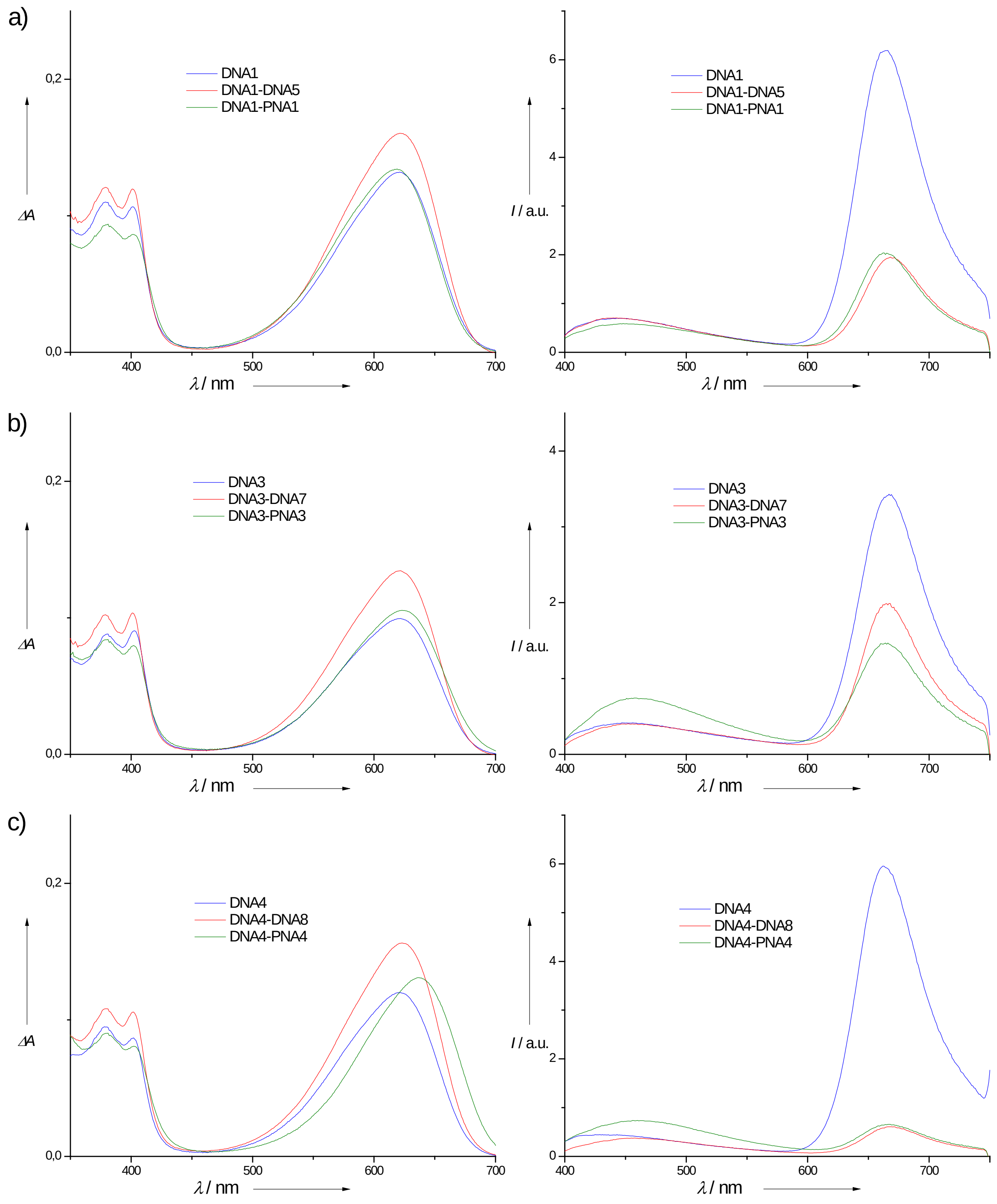

Figure S13. UV/Vis absorption (left) and fluorescence (right) for: a) single stranded **DNA1**, double stranded **DNA1-DNA5** and **DNA1-PNA1**; b) single stranded **DNA3**, double stranded **DNA3-DNA7** and **DNA3-PNA3**; c) single stranded **DNA4**, double stranded **DNA4-DNA8** and **DNA4-PNA4**; each 2.5 µM duplex, 10 mM Na-P_i buffer, pH 7, 250 mM NaCl, 20 °C, λ_{exc} 380 nm.
